# New Journals

**Published:** 1874-08

**Authors:** 


### NEW JOURNALS.

THE PSYCOLOGICAL AND MEDICO-LEGAL JOURNAL. Conducted by W. A. Hammond, M.D., assisted by T. M. B. Cross, M.D. New York: F. W. Christern. In monthly numbers of 72 large octavo pages. $5 per year.

We gladly welcome back to the journalistic tripod Dr. Hammond, who resumes, with the current number, the publication which he founded, and so ably conducted until its suspension some time ago. We trust his labors in his chosen field will be properly appreciated, and the publisher encouraged by a good, generous supscription list.

---

THE AMERICAN MEDICAL WEEKLY. E. S. Gaillard, M.D., editor and proprietor. Louisville, Ky. $2 per year.

In response to the demand for a journal making frequent visits, and filled with short and practical articles, this journal has been undertaken. It will not interfere in any way with the editor's larger work, the monthly. We heartily wish for the new candidate that professional favor and success which we believe it is destined to achieve.

---

SUPPLEMENT TO THE MEDICAL NEWS AND LIBRARY. Philadelphia: Henry C. Lea, publisher. Monthly. Pp. 48.

This publication takes the place of the Half Yearly Abstract, which has been suspended, and is conducted upon the same general plan, excepting that it is issued monthly, instead of semi-annually. This change, we believe, will be received with favor by the profession. It is certainly in the direction of progress.

